# Patient characteristics and admitting vital signs associated with coronavirus disease 2019 (COVID-19)–related mortality among patients admitted with noncritical illness

**DOI:** 10.1017/ice.2020.461

**Published:** 2020-09-15

**Authors:** Kenneth E. Sands, Richard P. Wenzel, Laura E. McLean, Kimberly M. Korwek, Jonathon D. Roach, Karla M. Miller, Russell E. Poland, L. Hayley Burgess, Edmund S. Jackson, Jonathan B. Perlin

**Affiliations:** 1 Clinical Services Group, HCA Healthcare, Nashville, Tennessee; 2 Department of Internal Medicine, Virginia Commonwealth University Medical Center, Richmond, Virginia

## Abstract

**Objective:**

To determine risk factors for mortality among COVID-19 patients admitted to a system of community hospitals in the United States.

**Design:**

Retrospective analysis of patient data collected from the routine care of COVID-19 patients.

**Setting:**

System of >180 acute-care facilities in the United States.

**Participants:**

All admitted patients with positive identification of COVID-19 and a documented discharge as of May 12, 2020.

**Methods:**

Determination of demographic characteristics, vital signs at admission, patient comorbidities and recorded discharge disposition in this population to construct a logistic regression estimating the odds of mortality, particular for those patients characterized as not being critically ill at admission.

**Results:**

In total, 6,180 COVID-19+ patients were identified as of May 12, 2020. Most COVID-19+ patients (4,808, 77.8%) were admitted directly to a medical-surgical unit with no documented critical care or mechanical ventilation within 8 hours of admission. After adjusting for demographic characteristics, comorbidities, and vital signs at admission in this subgroup, the largest driver of the odds of mortality was patient age (OR, 1.07; 95% CI, 1.06–1.08; *P* < .001). Decreased oxygen saturation at admission was associated with increased odds of mortality (OR, 1.09; 95% CI, 1.06–1.12; *P* < .001) as was diabetes (OR, 1.57; 95% CI, 1.21–2.03; *P* < .001).

**Conclusions:**

The identification of factors observable at admission that are associated with mortality in COVID-19 patients who are initially admitted to non-critical care units may help care providers, hospital epidemiologists, and hospital safety experts better plan for the care of these patients.

The novel severe acute respiratory coronavirus virus 2 (SARS-CoV-2) emerged in China in late 2019, quickly spread globally, and was officially declared a pandemic in early March 2020. The first identified case of novel coronavirus disease 2019 (COVID-19) in the United States occurred in Washington State in late January 2020; subsequently available postmortem studies have indicated that cases arrived weeks earlier in California. This disease has spread rapidly across the country, with clusters appearing in certain geographic locations. Based on genetic analysis of circulating virus strains, most cases in the United States, especially those on the East Coast, appear to have European origin.^1^ As of May 27, 2020, there have been 1,678,843 cases and 99,031 deaths reported in the United States.^2^ While there have been coordination efforts led at the federal and state levels, much of the knowledge related to the management of these patients within the inpatient environment has been isolated within individual facilities and regional systems.

Early descriptions of the patients in China showed initial symptoms of fever, cough, dyspnea, and diarrhea.^3–5^ Older patients with underlying comorbidities were at greater risk of dying. Lymphopenia was a common laboratory finding, as was pneumonia on chest x-ray; ground-glass opacity findings on chest computed tomography scan were observed in severe cases.^6,7^ A minority of cases were deemed critical. Deaths in China followed the appearance of severe sepsis with multiple-organ failure, findings confirmed in an early study of 24 ICU patients in Seattle.^8^ A larger ICU study from the Lombardy region of Italy showed the prevalence of older men with underlying cardiac disease and diabetes among those who required mechanical ventilation and high levels of positive end-expiratory pressure (PEEP).^9^

Preliminary studies have shown differences in the mortality rate and outcomes of COVID-19 patients worldwide.^10–14^ This may be due to intrinsic differences within the patient population, such as age and underlying comorbidities, different strains of the virus, or the type of care and resources available at different sites. Thus, more data are needed regarding the characteristics of patients with COVID-19 in the United States, particularly for those patients who are not critically ill at admission but who decompensate sometimes quickly over time.

Early identification of COVID-19–positive (COVID-19+) patients at risk of critical illness or poor outcomes may aid in the delivery of care and the optimization of resource use. The purpose of this study was to describe the demographic characteristics and vital signs on presentation of a more representative sample of COVID-19+ patients in the United States and to determine subsets of these by illness severity at admission. These data were used to model the odds of mortality for patients that present with noncritical illness at admission and to identify the factors observable at admission associated with mortality risk.

Based on data from our network of >180 affiliated acute-care facilities across the United States, we have created a detailed composite of the demographics and movement of COVID-19+ patients within the hospital environment. We studied the trajectory of patient movement from first presentation to general medical wards to critical care to placement on respirators and death as well as the return to less severe stages in those who recovered. The combination of patient demographics and vital signs at admission were used to estimate the odds of mortality, adjusted for comorbidities and disease severity. In total, this analysis provides information about potential predictors of COVID-19 mortality from a national data set among patients who present with noncritical illness at admission that can be used by clinicians, hospital epidemiologists, and hospital safety experts to better target resources and improve patient care.

## Methods

### Setting

This retrospective analysis was conducted using data collected by HCA Healthcare, a large healthcare system with >180 affiliated acute-care facilities among its >2,000 associated sites of care in 21 US states and the United Kingdom. Most of these facilities are medium-to-large community hospitals; collectively these facilities provide ~5% of hospital care in the United States.

This research was deemed exempt from institutional review board oversight in accordance with institutional policy.

### Data access and definitions

Data from clinical care activities at affiliated facilities are collected via the electronic health record (EHR) and collated into a central repository. This clinical data warehouse (CDW) aggregates data streams for retrospective analysis and use in real-time data decision-support tools to inform care.

Patient encounter data were collected from the CDW for patients with confirmed COVID-19 disease as of March 1, 2020, and discharge dates as of May 12, 2020, from affiliated facilities in the United States. COVID-19 status was determined by the presence of at least 1 documented positive detection of SARS-CoV-2 by RT-PCR; this included patients with positive results from outside of the affiliated system that were documented at admission or transfer. For patients with >1 documented encounter in the data set, such as those with a readmission or transfer from another affiliated facility, only the last encounter was included for analysis.

Patient trajectory was determined by location as of midnight on each day of the encounter. Patient characteristics were collected from the EHR. Basic demographics included age, sex, race, ethnicity, body mass index (BMI), self-reported smoking status (current smoker, former smoker, never smoker), and insurance status.

Patient vital signs at admission (eg, temperature, heart rate, oxygen saturation, respiratory rate) and comorbidities present on admission (eg, cancer, hypertension, congestive heart failure, cardiac arrhythmias, pulmonary circulation disorders, paralysis, chronic lung disease, HIV/AIDS, transplant, renal failure, liver disease, diabetes, extreme obesity) were also collected from the EHR. The highest patient severity was defined as the highest level of service a patient received during the encounter as follows: (1) no documentation of care within a critical care unit, (2) intensive care within a critical care unit but no required ventilator support; or (3) required ventilator support. Mortality was determined by a discharge status of “expired.” Comorbidities were determined using ICD-10 codes from present on admission diagnoses.

### Statistical analysis

Ridge regression (L2 regularization) for variable selection was conducted to determine patient demographic and clinical characteristics that had a statically significant effect on mortality for inclusion in the overall regression model. Variable selection was verified through univariate analysis.

The primary outcome of interest was in-hospital mortality. Logistic regression was used to estimate the covariate-adjusted association between the selected patient demographic and clinical characteristics and a discharge disposition of expired. Analysis of deviance was used to establish a metric for variable importance.

## Results

We identified 6,180 COVID-19+ patient encounters that had a recorded discharge status as of May 12, 2020. The median age was 63 years, and the population was 47.8% female. Overall, 980 patients (15.9%) required mechanical ventilation during their encounter. The average length of stay was 7.5 days. The overall mortality was 19.1% (1,181 patients). Of the patients that did not expire in the hospital, 63% (3,879) were discharged home. The remaining patients were discharged to hospice (158, 2.6%) or transferred to another affiliated facility (962, 15.6%). Approximately 1.2% (76) of patients were readmitted within seven days. Demographics for COVID-19+ patients by discharge disposition are presented in Table 1. Patients who expired tended to be older with more severe comorbidities.

**Table 1.** Demographic Characteristics and Comorbidities of COVID19+ Patient Encounters by Discharge Disposition

Variable	Expired	Did Not Expire	*P* Value
	(N = 1,181)	(N = 4,999)	
Age at admission, median y [IQR]	76.0 [67.0–84.0]	59.0 [46.0–72.0]	<.001
**Sex, no. (%)**			<.001
Female	510 (43.2)	2447 (48.9)	
Male	671 (56.8)	2552 (51.1)	
**Race, no. (%)**			<.001
White	669 (56.6)	2332 (46.6)	
Asian	58 (4.9)	206 (4.1)	
Black	265 (22.4)	1269 (25.4)	
Native American or Alaska Native	1 (0.1)	5 (0.1)	
Native Hawaiian/other Pacific Islander	3 (0.3)	6 (0.1)	
Other	148 (12.5)	995 (19.9)	
Unknown	37 (3.1)	186 (3.7)	
**Ethnicity, no. (%)**			<.001
Not Hispanic or Latino	907 (76.8)	3391 (67.8)	
Hispanic or Latino	231 (19.6)	1373 (27.5)	
Unknown	43 (3.6)	235 (4.7)	
**Insurance type, no. (%)**			<.001
Charity	1 (0.1)	29 (0.6)	
Commercial	195 (16.5)	1369 (27.4)	
Medicare	673 (57.0)	2013 (40.3)	
Military	0 (0)	26 (0.5)	
Other	24 (2.0)	200 (4.0)	
Public federal	143 (12.1)	1282 (25.6)	
Public nonfederal	141 (11.9)	48 (1.0)	
Self pay	4 (0.3)	32 (0.6)	
Body mass index [IQR]	28.6 [24.2–34.0]	29.3 [25.1–34.8]	.18
Missing, no. (%)	220 (18.6)	594 (11.9)	
**Smoking status, no. (%)**			<.001
Never smoked	400 (33.9)	3254 (65.1)	
Former smoker	177 (15.0)	769 (15.4)	
Smoker	20 (1.7)	238 (4.8)	
Unknown	283 (24.0)	479 (9.6)	
Missing	301 (25.5)	259 (5.2)	
Length of stay, median d [IQR]	6.00 [2.00–11.0]	5.00 [3.00–10.0]	.04
**Highest level of severity, no. (%)**			
Non-ICU	294 (24.9)	3660 (73.2)	<.001
ICU	292 (24.7)	954 (19.1)	
Mechanical ventilation	595 (50.4)	385 (7.7)	

Note. IQR, interquartile range; ICU, intensive care unit.

**Fig. 1.**
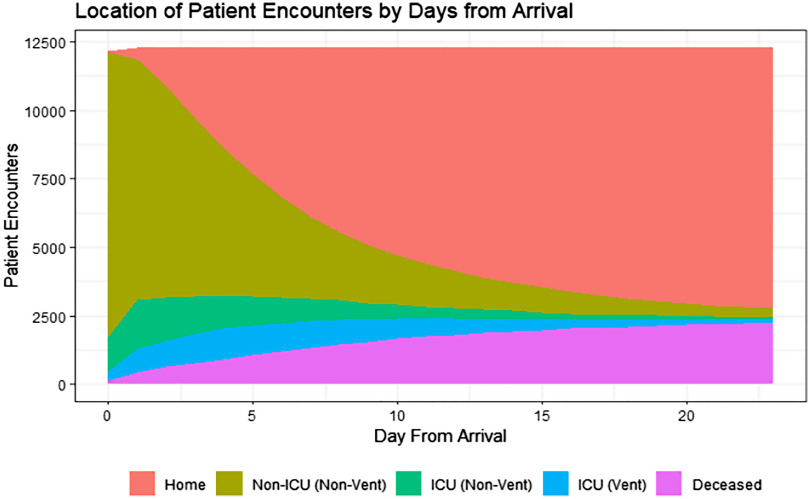
Trajectory of COVID-19+ patient encounters from admission to discharge. Number of patient encounters by day. Day of admission is represented as day zero. Locations are determined by midnight census. Home = discharged alive; Non-ICU (non-vent) = any location that does not provide critical care, no mechanical ventilation; ICU (non-vent) = any location that provides critical care, no mechanical ventilation; ICU (vent) = any location that provides critical care AND patient requires mechanical ventilation; diseased = discharged expired.

Of the total COVID19+ patient encounters, 4,808 of the total 6,180 (77.8%) were admitted directly to a medical/surgical unit with no documented care within an intensive care unit (or a unit designated to provide an equivalent level of critical care) or mechanical ventilation within 8 hours of admission. Approximately 16% of COVID+ patient encounters (n = 984) were admitted directly (within 8 hours of admission) to an intensive care unit or a unit providing the equivalent level of critical care support, while ~6% (388) were on mechanical ventilation at admission (Fig. 1).

For patients initially admitted to a medical-surgical unit, the overall mortality rate was 12.8% (617 of 4,808). For patients admitted directly to intensive care, the mortality rate was 32.4% (319 of 984); for patients placed on mechanical ventilation immediately upon admission, mortality was 63.1% (245 of 388). Thus, patients initially admitted to a medical/surgical unit accounted for over half (52.2%) of the total mortality (617 of 1,181).

Overall, ~10.3% (636 of 6,180) of patients presented at admission with a temperature above 38 C, and 5,211 of 6,180 patients (84.3%) presented with an O_2_ saturation >90%. Of 6,180 patients, 3,346 (54%) required supplemental oxygen at admission; however, these data are as documented by nursing and may exclude those known to be on a ventilator or those with a missing response (1,728, 28%). Approximately 14% (861) of patients presented with a respiratory rate >24 breaths per minute.

We identified potential risk factors for patient mortality using a logistic regression model. In a preliminary model, a large driver of mortality was severity at admission, namely the need for ventilator support and/or ICU care at admission. For this reason, and because the majority of the population was initially admitted to a non-critical care unit (Fig. 1), we limited our regression analyses to only those patients who were admitted to a non-ICU environment (no ICU or ventilator support within 8 hours of admission). As mentioned, >50% of the total mortality came from this group of patients.

We identified 4,808 COVID-19+ patients admitted to non-ICU environments. Demographic information for these patients is presented in Table 2. The mortality rate for this population was 12.8% (n = 617). The odds ratios of mortality were estimated using a model that adjusted for patient demographics, admission vitals, and comorbidities. The most common comorbidities present on admission were hypertension (n = 3,867, 63.4%), fluid and electrolyte disorders (n = 3,034, 49.1%), diabetes (n = 2,417, 39.1%), obesity (n = 1,492, 24.1%), chronic pulmonary disease (n = 1,279, 20.7%), renal failure (n = 1,182, 19.1%), and cardiac arrhythmia (n = 1,107, 17.9%).

**Table 2.** Demographic Characteristics and Comorbidities of COVID19+ Patient Encounters Admitted to Noncritical Care Units, by Discharge Disposition

Variable	Expired (N = 617)	Did Not Expire (N = 4191)	*P* Value
Age at admission, median y [IQR]	78.0 [69.0–86.0]	60.0 [46.0–72.0]	<.001
**Sex, no. (%)**			.006
Female	286 (46.4)	2,096 (50.0)	
Male	331 (53.6)	2,095 (50.0)	
**Race, no. (%)**			<.001
White	365 (59.2)	1,959 (46.7)	
Asian	36 (5.8)	174 (4.2)	
Black	132 (21.4)	1,056 (25.2)	
Native American or Alaska native	0 (0)	3 (0.1)	
Native Hawaiian/other Pacific islander	3 (0.5)	5 (0.1)	
Other	66 (10.7)	844 (20.1)	
Unknown	15 (2.4)	150 (3.6)	
**Ethnicity, no. (%)**			<.001
Not Hispanic or Latino	478 (77.5)	2,851 (68.0)	
Hispanic or Latino	119 (19.3)	1,162 (27.7)	
Unknown	20 (3.2)	178 (4.2)	
**Insurance type, no. (%)**			<.001
Charity	1 (0.2)	22 (0.5)	
Commercial	84 (13.6)	1,137 (27.1)	
Medicare	369 (59.8)	1,699 (40.5)	
Military	0 (0)	21 (0.5)	
Other	19 (3.1)	176 (4.2)	
Public federal	65 (10.5)	1,075 (25.7)	
Public non federal	78 (12.6)	35 (0.8)	
Self pay	1 (0.2)	26 (0.6)	
BMI [IQR]	27.5 [23.5–33.0]	29.3 [25.2–34.7]	.19
Missing	106 (17.2%)	571 (13.6%)	
**Smoking status, no. (%)**			<.001
Never smoked	248 (40.2)	2,798 (66.8)	
Former smoker	111 (18.0)	655 (15.6)	
Smoker	14 (2.3)	199 (4.7)	
Unknown	135 (21.9)	363 (8.7)	
Missing	109 (17.7)	176 (4.2)	
Length of stay, median d [IQR]	6.00 [3.00–12.0]	5.00 [3.00–9.00]	.001
**Highest level of severity, no. (%)**			<.001
Non-ICU	294 (47.6)	3,660 (87.3)	
ICU	79 (12.8)	354 (8.4)	
Mechanical ventilation	244 (39.5)	177 (4.2)	

Note. IQR, interquartile range; ICU, intensive care unit.

**Fig. 2.**
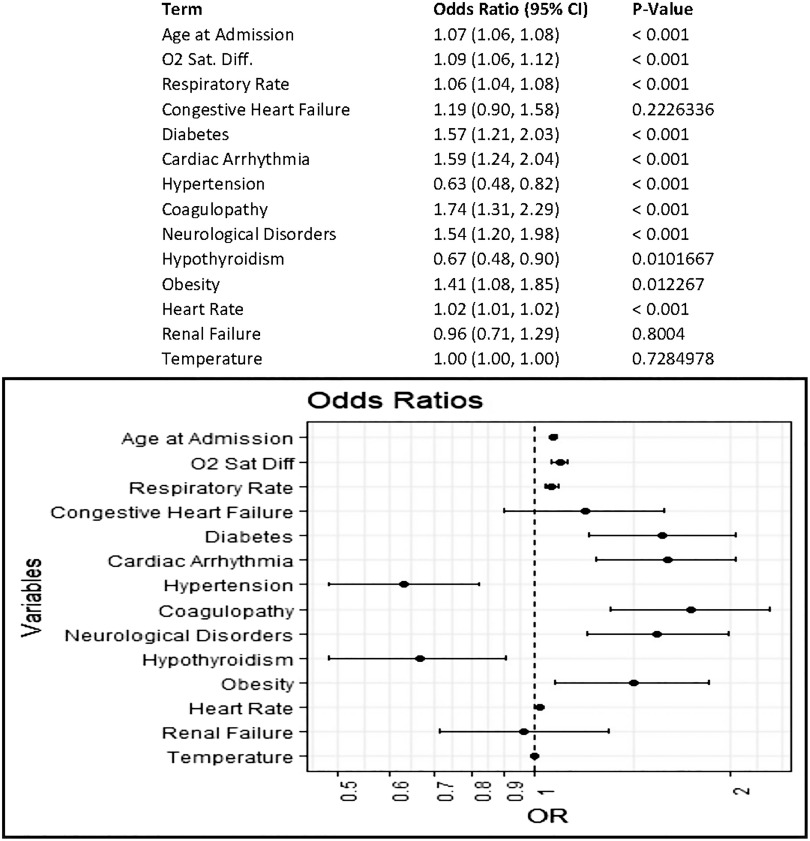
Odds of mortality in COVID19+ patient encounters initially admitted to noncritical care units. Multivariable logistic regression to estimate covariate-adjusted association of patient characteristics and vital signs at admission with a discharge disposition of expired. Comorbidities represent those indicated as present on admission in the final discharge codes. Vital sign measurements represent the first measurement recorded at admission; measurement must have occurred within 8 hours of admission.

Among patients admitted to non–critical care units, the largest driver of mortality was age at admission, which accounted for ~62% of the variance in the model (OR, 1.07; 95% CI, 1.06–1.08; *P* < .001). Decreased oxygen saturation (difference from 100%), increased respiratory rate, and diabetes were also associated with increased odds of mortality; these accounted for 13.5%, 6.4%, and 2.7% of the deviance from the null model, respectively (Fig. 2).

## Discussion

Using a large, geographically diverse composite of hospitalized patients with COVID-19 across the United States, we have identified patient characteristics and vital signs at presentation that are associated with mortality. As of May 12, 2020, we have identified 6,180 COVID-19+ patients discharged from facilities affiliated with a large healthcare system in the United States. Most of these patients were admitted to non–critical care units. Among COVID-19+ patient encounters admitted with noncritical illness, we detected increased odds of mortality associated with increased age, diabetes, and lower oxygen saturation at admission. Because the population served by this healthcare system is highly diverse and representative of the US population in general, these results are likely to be generalizable and represent a potential constellation of predictors with which to assess patient risk and inform care.

Admission directly to an ICU or placement on mechanical ventilation at admission is, as expected, associated with greater risk of mortality. However, as mentioned, a much greater proportion of COVID-19+ patients are admitted to non–critical-care units. Although most patients will recover, many will eventually require more advanced care or will ultimately expire. In our data set, ~50% of deaths occurred patients initially admitted to non–critical-care units. The ability to identify patients at risk for worse outcomes based on easily observable characteristics and vital signs early on could aid providers in the targeting of treatment and monitoring of at-risk patients, and making sure resources are available for transfer to the ICU.

Our large data set allowed us to track the trajectory of COVID-19 patients admitted to hospitals in the United States. A COVID-19+ patient spends an average of 5 days in the hospital after initial entry. On average, 4 of these days will be spent in a non-ICU location (emergency department or medical-surgical unit). If the patient goes to the ICU (nonventilated), their expected length of stay in the ICU is 5 days. If the patient is ventilated, their expected length of stay in that location is 9 days.

After adjusting for all other covariates, age remained the largest influence on the odds of mortality, with each year of age associated with a 7% increase in the odds of mortality. This finding aligns with results from previous studies that showed increased mortality among older patients.^15,16^ Most patients in our data set did not present at admission with a fever, but lower oxygen saturation and higher respiratory rate were associated with increased odds of mortality. Thus, patients with these characteristics may benefit from being more closely monitored. Among patients initially admitted to a non–critical-care unit who ultimately expired, the subgroup that was never admitted to an ICU or placed on mechanical ventilation tended to be older with lower oxygen saturation at admission. Thus, this indicates that there is the potential for further study of which non–critically ill COVID-19 patients would benefit from more intensive monitoring or a higher level of care at admission.

Of all the comorbidities analyzed, diabetes had the most influence on the odds of mortality. Patients with diabetes were ~9 times as likely as patients without diabetes to expire. This finding is in line with previous reports of increased mortality in this population.^17–19^ Importantly, our analysis, like many others, only takes into consideration the presence of a known diabetes diagnosis. We did not account for how well the blood sugar levels were controlled prior to the COVID-19–related admission, the presence of hyperglycemia without a diabetes diagnosis, or the glycemic control variation within the hospital. Thus, certain high-risk patients in the medical-surgical environment might benefit from blood-glucose monitoring comparable to that which occurs in the ICU. We are currently exploring these factors within this data set for a future analysis.

The most common comorbidities in this population reflected some of the most common comorbidities in the US population, namely hypertension, diabetes, obesity, chronic pulmonary disease, and renal failure. This finding reflects the generalizability of our patient population. Notably, our healthcare system is heavily represented in the southern United States, with larger representations from Florida and Texas; therefore, our results may differ from those found in other areas of the country, such as New York or the West Coast. However, facilities in our system are largely representative of the US healthcare facilities as a whole; they include small and medium-sized community hospitals as well as larger facilities. By many measures, the admitted population in affiliated facilities is more diverse than the hospitalized population on average. Thus, our findings offer the opportunity for greater generalization.

In our overall population, there were statically significant differences in mortality based on race, ethnicity, and insurance status. This finding mirrors similar findings in other studies that have suggested an increased rate of mortality and critical illness among certain racial and ethnic groups or within underserved populations. However, when we adjusted for all comorbidities, vital signs at admission, and other patient characteristics among patients admitted to a non–critical-care unit, we no longer detected a strong association of race, ethnicity, or insurance status with higher mortality risk. This finding suggests that there may be a more complicated interaction among these factors and other variables that could influence the risk of mortality. For instance, under- or uninsured patients may be less likely to seek care at an earlier stage of the disease; therefore, they may be admitted directly to critical care or other intensive treatment when they do eventually seek care. Also, our data on patient race and ethnicity have few missing values, and our data are dependent on the patient’s comfort level with self-identification of this information at admission or, if the patient or family members are unable to communicate, a subjective opinion by hospital personnel. In addition, certain racial and ethnic groups tend to be younger than the US population on average in the areas serviced by our affiliated facilities. As age is by far the greatest contributor to mortality, this age distribution could potentially skew our findings.

Two interesting findings deserve additional comment. We found that hypertension and, to a lesser extent, hypothyroidism noted as present on admission from *International Classification of Disease, Tenth Revision* (ICD-10) codes at discharge were associated with decreased odds of mortality. Whether the observed reductions in the odds of mortality were due to the diseases themselves or to the medications used to treat these disorders is currently under investigation. For instance, the use of angiotensin-converting enzyme (ACE) inhibitors and angiotensin-receptor blockers (ARBs) may modulate the receptors that bind to SARS-CoV-2, thereby altering virus uptake. These medications are commonly used by patients with hypertension, so this factor could underlie the reduction in the odds of mortality we observed. However, studies about these medications in COVID-19 patients are not yet conclusive.^20,21^ Similarly, few studies have investigated hypothyroidism and COVID-19, but there may be potential for future research in this area as new observations emerge.^22^

Similar to any retrospective analysis of real-world data, our study was limited by the variation in local practices and the completeness of the data set. Our data were drawn from information collected in the course of care; thus, a certain level of missing data is to be expected. This is especially true in a pandemic situation where regular staff and procedures may not be in place. Overall, we saw a moderate level of missing data in our patient demographics and vital signs. We performed additional analyses to ensure that these data were missing at random and were due to systematic facility- or patient-level discrepancies. We also imputed missing values where appropriate for modeling purposes. Therefore, we are confident that our data set and the associated results are a fair and valid representation of the hospitalized COVID-19+ patient population.

We have provided a detailed composite of the characteristics of COVID-19+ patient encounters in the United States, and we have identified characteristics observable at admission that are associated with mortality. To our knowledge, this is the first investigation of COVID-19+ patients that present with noncritical illness, which is how most COVID-19+ patients are initially admitted, and the observable factors at admission that are associated with a greater risk of mortality. These findings can aid providers, hospital epidemiologists, and hospital safety experts in the identification of COVID-19+ patients at high risk immediately upon admission, and they may help target monitoring or a higher level of service that could reduce possible progression to severe disease or death.
